# Ultrahigh Oxygen Reduction Reaction Electrocatalytic Activity and Stability over Hierarchical Nanoporous N-doped Carbon

**DOI:** 10.1038/s41598-018-21213-3

**Published:** 2018-02-12

**Authors:** Zeyu Li, Qiuming Gao, Weiwei Qian, Weiqian Tian, Hang Zhang, Qiang Zhang, Zhengping Liu

**Affiliations:** 1 Key Laboratory of Bio-inspired Smart Interfacial Science and Technology of Ministry of Education, Beijing Key Laboratory of Bio-inspired Energy Materials and Devices, School of Chemistry, Beihang University, Beijing, 100191 P. R. China; 2 Institute of Polymer Chemistry and Physics of College of Chemistry, BNU Lab of Environmentally Friendly and Functional Polymer Materials, Beijing Normal University, Beijing, 100875 P. R. China

## Abstract

Hierarchical nanoporous N-doped carbon ZNC-1000 was prepared by facile pyrolysis of well-designed nanosized ZIF-8 precursor with optimized reaction temperature and time. It possesses large surface areas leading to sufficient exposed electrochemical active sites. Meanwhile, its moderate graphitization degree and suitable nanosized hierarchical porosity distributions would lead to the sufficient interaction between O_2_ and the electrocatalyst surface which would benefit the transports of electrons and the electrolyte ions for ORR. As an electrocatalyst for oxygen reduction reaction, the ZNC-1000 presents a better catalytic property than the commercial Pt/C with 6/1 mV positive shifts for onset/half-wave potentials and 1.567 mA cm^−2^ larger for limiting current density respectively. The stability of ZNC-1000 is also much better than that of Pt/C with negative shifts of 0/−2 mV (vs 5/31 mV) for onset/half-wave potentials and 6.0% vs 29.2% loss of limiting current density after 5000 cycles of accelerated durability test, as well as the relative current of 87.5% vs 40.2% retention after 30,000 s continuous chronoamperometric operation.

## Introduction

Oxygen reduction reaction (ORR) is one of the most important reactions in the fuel cells, metal-O_2_ batteries and so forth, since the naturally slow kinetics of the reduction reaction on cathode comparing with the anodic oxidation reaction, which makes it urgent for the research of high performance ORR electrocatalysts^1–5^. Even though the well-known Pt-based electrocatalysts are active for the ORR, their inherent characteristics such as the limited resource, high cost, *etc*. make them unfit for the large-scale application. Recently, diverse types of non-precious metal ORR electrocatalysts were studied such as transition metal oxides^6,7^, nitrides^8^, carbides^9^ and chalcogenides^10^. Additionally, the non-metal N, P, S and B elements doped carbonaceous materials were also developed^11–17^. The different electronegativities and atom sizes of the non-metal heteroatoms could lead to the alteration of the electron densities, resulting in formation of the active centers for ORR. Meanwhile, the non-metal heteroatoms doped in the carbonaceous framework could contribute to the improvement of the electrochemical stability^18^.

Metal-organic frameworks (MOFs), with the crystalline structure of metal ions or clusters linked together by N-involved organic linkers, could be an ideal precursor of transition metal-nitrogen-carbon composite due to their different structures, high surface areas and large pore volumes. Besides, they can work as both a sacrificial template and a secondary carbon precursor and form the nanoporous structure during the pyrolysis process. Zeolitic imidazolate frameworks (ZIFs) as a subclass of MOFs have been used as the precursors for ORR because of the high surface areas and the homogeneous distribution of heteroatoms of N and transition metal which would provide valid active sites. ZIF-8 was selected as the precursor due to its cage-type pore structure with large pore cavities of 1.16 nm connected by small pore apertures of 0.34 nm, which would form N-doped porous carbon after Zn evaporation during carbonization process^19–27^. However, further optimization of the element compositions, bonding types as well as pore structures and/or textures of the resultant porous carbonaceous electrocatalysts are still needed to enhance the ORR electrocatalytic properties. Herein, one kind of well-designed nanosized MOF ZIF-8 precursor was obtained and utilized to prepare the hierarchical nanoporous N doped carbon. The optimized ZNC-1000 material possessing of favourable doping N element and its bonding types in the carbonaceous structure, large surface areas and suitable porosity distributions, as well as moderate graphitization degree, presented an amazing high ORR electrocatalytic performance.

## Results and Discussion

### Structural characterization

**Figure 1:**
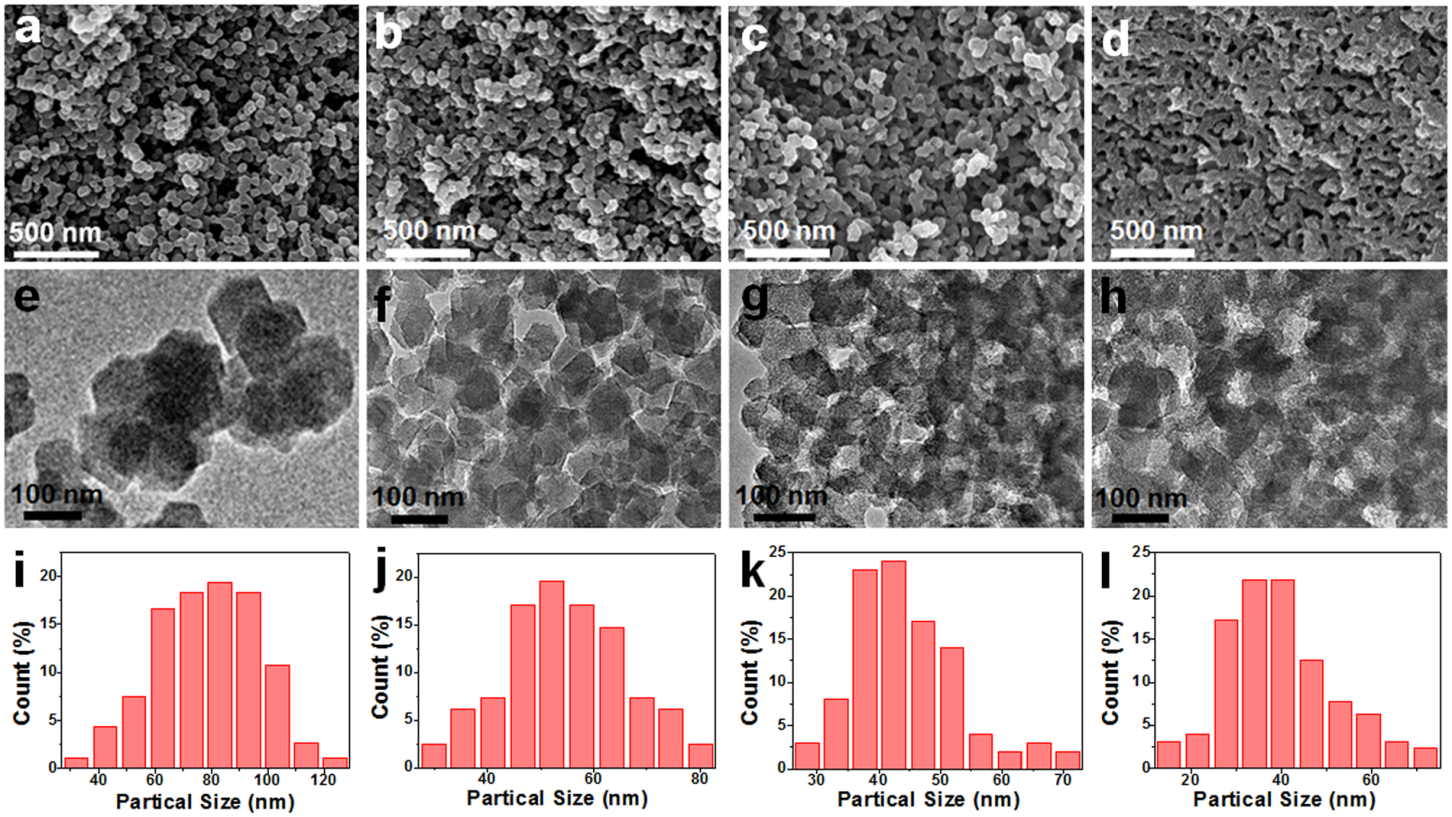
SEM and TEM images with their particle size distributions of ZIF-8 (**a,e,i**), ZNC-900 (**b,f,j**), ZNC-1000 (**c,g,k**) and ZNC-1100 (**d,h,l**).

Well-designed ZIF-8 precursor was synthesized by combining ultrasonic dispersion treatment with room temperature solution reaction in the ethanol solution including 2-methylimidazole and Zn(NO_3_)_2_·6H_2_O. The N-doped carbonaceous ZNC-900, ZNC-1000 and ZNC-1100 materials were obtained from pyrolysis of the ZIF-8 precursor with the even particles size of about 78.6 nm from the observation on the scanning electron microscope (SEM) and transmission electron microscopy (TEM) images in Fig. 1a,e. After the annealing treatment in N_2_ atmosphere, the organic ligand 2-methylimidazole of ZIF-8 was transformed into N-doped nanoporous carbon and the ZNC-900, ZNC-1000 and ZNC-1100 particles are uniform with the sizes of about 54.6, 44.6 and 39.8 nm, respectively, based on the observation from the SEM (Fig. 1b–d and Fig. S1a,d,g) and TEM images (Fig. 1f–h and Fig. S1b,e,h). Compared with that of ZIF-8, the particles of ZNC-900, ZNC-1000 and ZNC-1100 shrank obviously step by step with the increased pyrolysis temperature from 900 to 1100 °C (Fig. 1i–l). The small particle sizes may lead to good diffusion of O_2_ in the pores using as the ORR electrocatalyst. Besides, the particles cross-linked increasingly and the boundary of the particles became more and more blurred with the enhanced pyrolysis temperature from 900 to 1100 °C. The connection between the particles would be beneficial for improvement of the conductivity. High-resolution TEM (HRTEM) images in (Fig. S1c,f,i) show that the small nanopores are very homogeneous and the graphitization degree increases with the more and more obviously crystallized fringes from ZNC-900, ZNC-1000 to ZNC-1100. The N content decreased from 13.38 to 4.65 at.% for ZNC-900 and ZNC-1000 and only 1.83 at.% was determined for ZNC-1100 based on the EDX analyses (Table S1). The Zn content also decreased from 3.03 to 0.63 and then to 0.19 at.% for ZNC-900, ZNC-1000 and ZNC-1100, since the Zn^2+^ ions could be reduced and evaporated leaving the nanopores in the carbonaceous frameworks at the high temperature with bit left linked by Zn-N bonds^28^.

**Figure 2:**
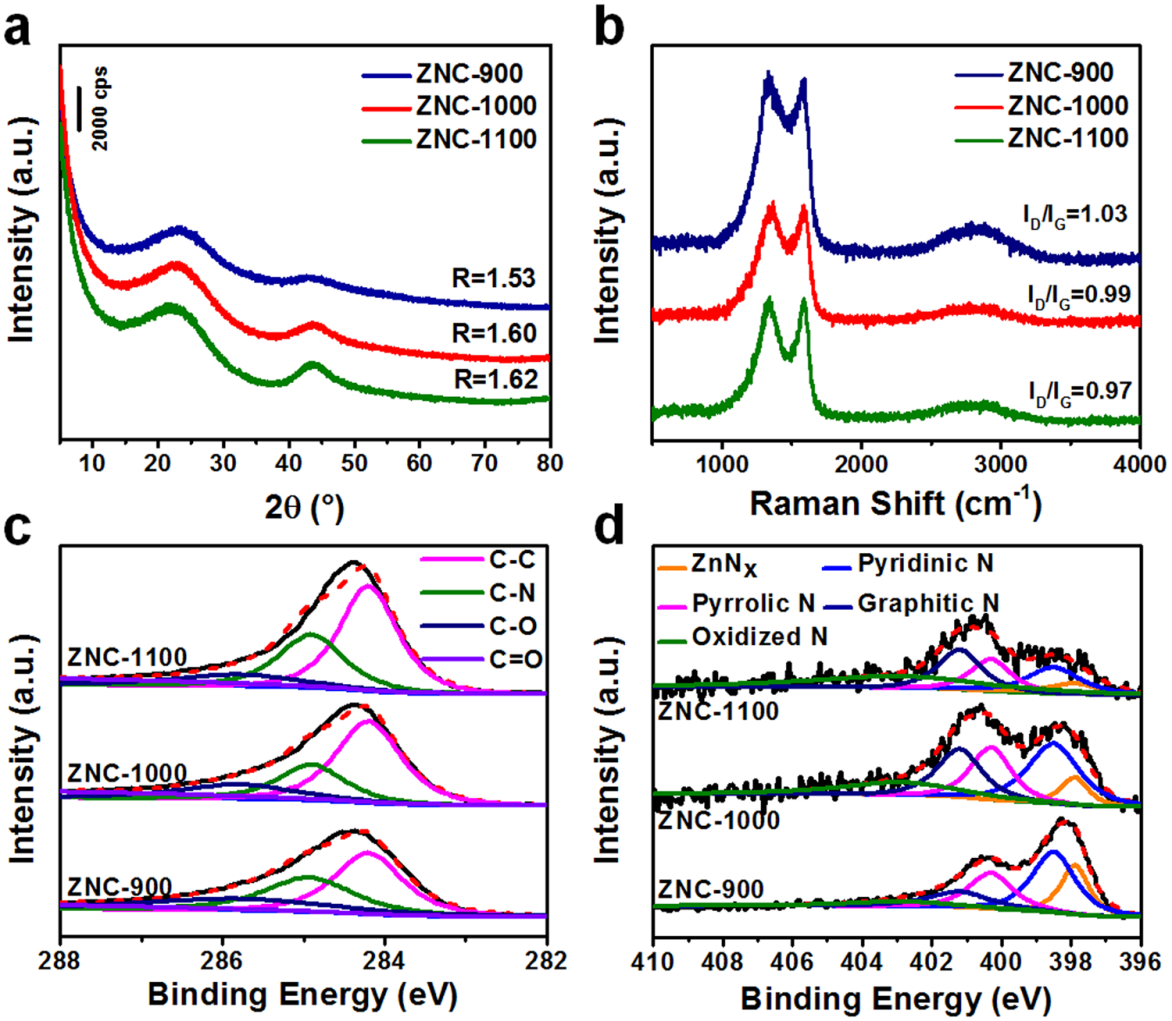
XRD patterns (**a**), Raman spectra (**b**), and high resolution XPS spectra of C 1 s (**c**) and N 1 s (**d**) of ZNC-900, ZNC-1000 and ZNC-1100.

X-ray diffraction (XRD) patterns (Fig. 2a) were determined to characterize the microstructures of ZNC-900, ZNC-1000 and ZNC-1100, where two broad XRD peaks at about 26.3 and 44° were found for all the samples corresponding to the (002) and (100)/(101) diffractions of carbon, respectively^29^. The graphitization degree can be indicated by the R value, which is defined as the ratio of height of the (002) diffraction peak (B) to background (A)^30^. The R value increased from 1.53, 1.60 to 1.62 for ZNC-900, ZNC-1000 and ZNC-1100 indicating their gradually enhanced graphitization degree in agreement with the HRTEM image observation. The Raman spectroscopies of ZNC-900, ZNC-1000 and ZNC-1100 (Fig. 2b) display two peaks assigned to D-band (1354 cm^−1^) for defective sp^3^-phase and G-band (1588 cm^−1^) for ordered sp^2^-phase, respectively^31,32^. The integral ratio I_D_/I_G_ for ZNC-900, ZNC-1000 and ZNC-1100 decreased from 1.03, 0.99 to 0.97 indicating to a gradually enhanced graphitization degree, which may lead to a rising electronic conductivity thus benefits for the electron transport during the ORR process. However, the decreased doped N content may reduce the electrocatalytic active sites for ORR. So, the middle 1000 °C became the optimal pyrolysis temperature and ZNC-1000 performed the best ORR electrocatalytic activity as described below.

X-ray photoelectron spectroscopy (XPS) survey scan spectra of ZNC-900, ZNC-1000 and ZNC-1100 were measured and shown in Fig. S2 in which only C, N, O and Zn elements presented (Table S2) and both the N and Zn contents decreased with increase of the pyrolysis temperature in consistent with the EDX analyses. The C 1 s high resolution XPS spectra of the samples can be seen in Fig. 2c with the peaks attributed to the C-C, C-O, C=O and C-N bonds. Figure 2d showed their N 1 s high resolution XPS spectra with pyridinic N (398.5 eV), pyrrolic N (400.3 eV), graphitic N (401.2 eV), oxidized N (402.9 eV) and Zn-N (397.9 eV) bonds^33^. With increasing of the pyrolysis temperature from 900 to 1100 °C, the contents of pyridinic N decreased from 37.5 to 19.8% and the graphitic N increased from 13.7 to 25.0% due to the conversion from pyridinic N to graphitic N^29^. The highest content sum (54.7%) of pyridinic and graphitic N in ZNC-1000 among the samples (Table S2) indicates that these bond types are effective for the ORR electrocatalysis based on their shared electrons from the delocalized π systems. Besides, nitrogenation and porosity can jointly influence the ORR activities^34^.

**Figure 3:**
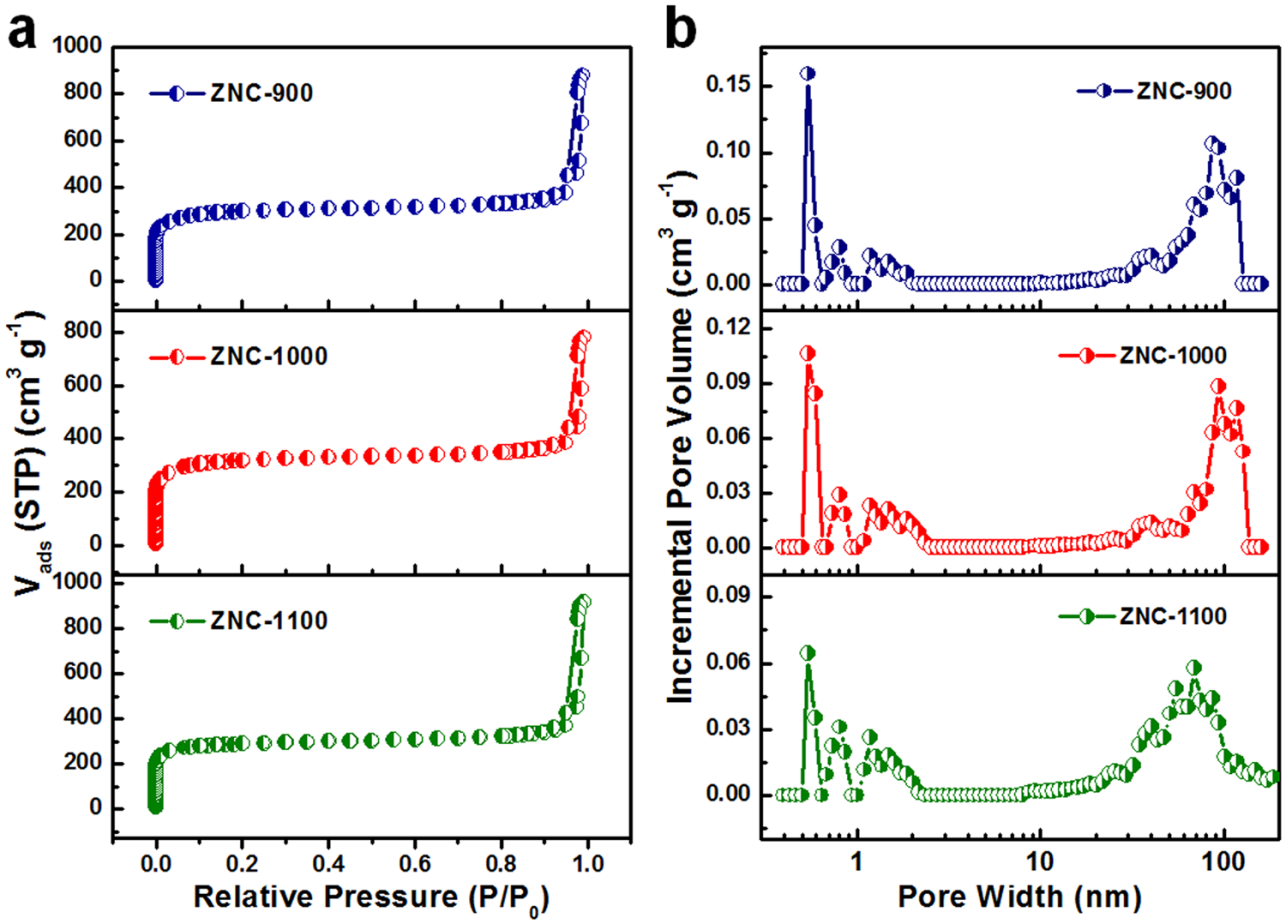
N_2_ sorption isotherms (**a**) and porosity distributions (**b**) of ZNC-900, ZNC-1000 and ZNC-1100.

The pore structures and textures of ZNC-900, ZNC-1000 and ZNC-1100 were confirmed by the N_2_ adsorption-desorption measurements (Fig. 3a,b). All samples possessed mixed Type-I and -IV isotherms with obvious sharpness at low pressure (P/P_0_ < 0.1), indicating the existence of abundant micropores. The porosity distributions (Table S3) showed hierarchical porous characteristics with plenty of micropores and certain amount of meso/macropores, which inherited from the ZIF-8 precursor and/or caused by the evaporation of Zn and N atoms. Since the micropores may be beneficial for the adsorption of O_2_ on the catalyst as the Yeager model (bridge model) with the tendency of 4e-dominated process for ORR than the Pauling model (edge-on adsorption) with the tendency of 2e-dominated process because of the limited spatial effect. Among them, ZNC-1000 presented the largest BET surface area of 1205 m^2^ g^−1^ and micropore surface area of 1035 m^2^ g^−1^, providing the largest effective catalytic active surface for ORR. Besides, the certain amount of meso/macropores in ZNC-1000 could be beneficial for the transport of the electrolyte ions. Thus, the suitable hierarchical pore structure and texture of ZNC-1000 may lead to the outstanding electrocatalytic activity for ORR.

### Electrochemical ORR evaluations

**Figure 4:**
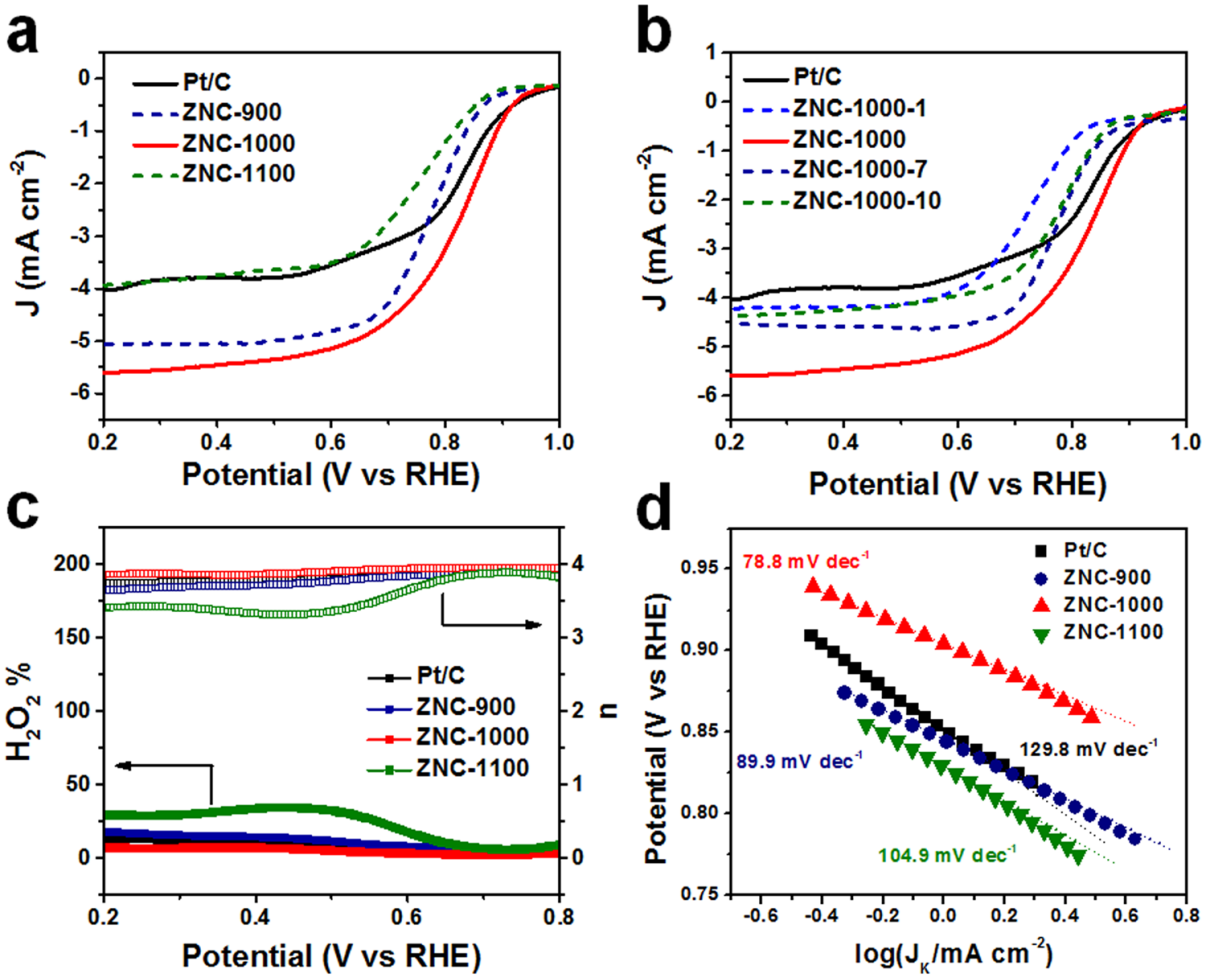
(**a,b**) The polarization curves of Pt/C and ZNCs at the rotating rate of 1600 rpm in O_2_-saturated 0.1 M KOH, (**c**) the peroxide yield with regard to the total oxygen reduction products and the calculated electron transfer number of Pt/C and ZNCs from RRDE and (**d**) the Tafel plots for Pt/C and ZNCs at the rotating rate of 1600 rpm.

The electrochemical activities of ZNC-900, ZNC-1000 and ZNC-1100 as well as the reference state of art commercial Pt/C catalyst with 20 wt.% Pt were evaluated by cyclic voltammetry (CV) measurements in the N_2_ and O_2_ saturated 0.1 M KOH electrolytes (Fig. S3 and Table 1). The cathodic peak could be obtained in saturated O_2_ in contrast to that in saturated N_2_ without peak for each sample. An excellent peak with the position at 0.865 V (vs reversible hydrogen electrode (RHE)) was observed for ZNC-1000, which is more positive than that of ZNC-900 (0.824 V) and ZNC-1100 (0.764 V). Both ZNC-1000 and ZNC-900 possessed superior peak positions to that of Pt/C (0.799 V). Further linear sweep voltammetry (LSV) measurements were carried out at 10 mV s^−1^ in O_2_ saturated 0.1 M KOH electrolyte to confirm the electrocatalytic activities (Table 1). The enhanced current densities could be observed for ZNC-900, ZNC-1000 and ZNC-1100 and Pt/C with increase of the rotation rate from 400 to 2000 rpm (Fig. S4). The LSV curves of the samples at a rotation rate of 1600 rpm are shown in Fig. 4a for comparison. The onset potential of ZNC-1000 presented a much more positive position at 0.915 V vs RHE than that of Pt/C (0.909 V), ZNC-900 (0.859 V) and ZNC-1100 (0.866 V). Meanwhile, ZNC-1000 possessed the most positive half-wave potential with the position at 0.813 V among them (vs 0.771 V for ZNC-900 and 0.748 V for ZNC-1100), which is also superior to that of Pt/C (0.812 V). The limiting current density of ZNC-1000 could reach 5.599 mA cm^−2^, which is much higher than that of Pt/C (4.032 mA cm^−2^) as well as ZNC-900 (5.056 mA cm^−2^) and ZNC-1100 (3.936 mA cm^−2^). Accordingly, the electrochemical catalytical performances of ZNC-1000-1, ZNC-1000-7 and ZNC-1000-10 with different pyrolysis time were evaluated in Fig. 4b, which were lower than that of ZNC-1000. Besides, rotating ring and disk electrode LSV measurement was utilized to expose the reaction pathway by detecting the H_2_O_2_ content as the intermediate during the ORR process (Fig. 4c). The H_2_O_2_ yield calculated from the equation at 0.2 V vs RHE was as low as 7.1% for ZNC-1000, which is apparently lower than that of Pt/C (13.2%), ZNC-900 (18.0%) and ZNC-1100 (29.4%). Thus, ZNC-1000 presented the most approximate four electron transfer ORR process with the electron transfer number of 3.86 at 0.2 V vs RHE, comparing with that of Pt/C (3.74), ZNC-900 (3.64) and ZNC-1100 (3.41).

**Table 1:** The ORR catalytic activities of the commercial Pt/C and ZNCs before and after ADT of 5000 cycles in O_2_-saturated 0.1 M KOH.

Sample	Peak potential (V vs RHE)	Onset Potential (V vs RHE)		Half-wave Potential (V vs RHE)		Limiting Current Density (mA cm^−2^)		
		Before ADT	After ADT	Before ADT	After ADT	Before ADT	After ADT	% loss
Pt/C	0.799	0.909	0.904	0.812	0.781	4.032	2.854	29.2%
ZNC-900	0.824	0.859	—	0.771	—	5.056	—	
ZNC-1000	0.865	0.915	0.915	0.813	0.815	5.599	5.264	6.0%
ZNC-1100	0.764	0.866	—	0.748	—	3.936	—

In order to understand the kinetic properties of ORR, the Tafel slopes for the commercial Pt/C and the ZNCs are shown in Fig. 4d from the linear plots of LSV with the rotating rate of 1600 rpm. In the region at low overpotential, where the overall ORR speed was determined by the surface reaction rate, the Tafel slope of ZNC-1000 presented a much lower value of 82.1 mV dec^−1^ than that of Pt/C (129.2 mV dec^−1^) and the other two samples (89.9 mV dec^−1^ for ZNC-900 and 104.9 mV dec^−1^ for ZNC-1100), indicating the fastest electron transfer rate of ZNC-1000 for ORR^33^. To further understanding the process of ORR, activation energy (E_a_) was evaluated by the following Arrhenius equation^35^ in the temperature reign of 283 to 333 K (Fig. S5): $\partial$(logJ_K_)/$\partial$(1/T) = ΔE_a_/2.3 R, where J_K_ was the kinetic current density (mA cm^−2^), T was the reaction temperature (K) and R was the universal gas constant (8.314 J mol^−1^ K^−1^). And η = 0.3 V vs RHE was chosen to calculate^36^. The ΔE_a_ values of ZNC-1000 and Pt/C were 28 and 32 kJ mol^−1^, respectively. The lower activation energy of ZNC-1000 than that of Pt/C indicates the higher electrocatalytic activity towards the reduction of O_2_ to H_2_O.

**Figure 5:**
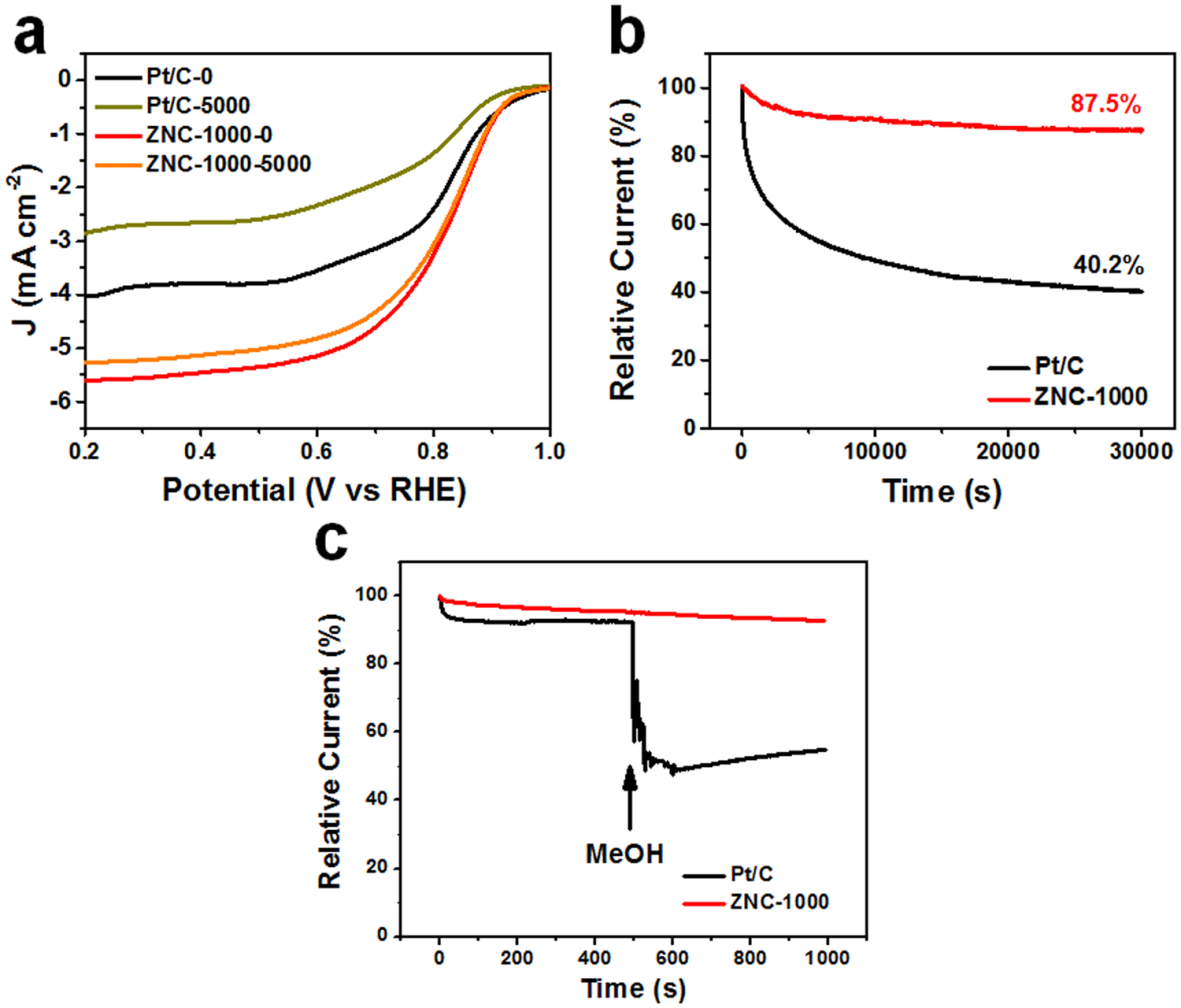
(**a**) The polarization curves before and after the ADT of 5000 cycles, (**b**) CA responses, and (**c**) the tolerance for methanol with 3 M methanol added into the O_2_-saturated 0.1 M KOH during the CA response of 500 s of Pt/C and ZNC-1000.

The stability of ZNC-1000 was evaluated by accelerated durability tests (ADT) and chronoamperometric (CA) measurements (Table 1). The better stability of ZNC-1000 than that of Pt/C can be found from the ADT measurements (Fig. 5a). After 5000 cycles of ADT at the scan rate of 100 mV s^−1^, the negative shifts of 0 mV for the onset potential and −2 mV for the half-wave potential of ZNC-1000 were seen contrast to that of Pt/C (5 and 31 mV negative shifts for the onset and half-wave potential, respectively). The limiting current density of ZNC-1000 also decreased slightly of 6.0% (from 5.599 to 5.264 mA cm^−2^), which is much better than that of Pt/C of 29.2% (from 4.032 to 2.854 mA cm^−2^). After 30,000 s of continuous CA operation (Fig. 5b), the relative current of ZNC-1000 maintained about 87.5% contrast to only 40.2% retention of Pt/C. Moreover, the test of the toleration for methanol is shown in Fig. 5c. A significant current decrease could be observed for Pt/C upon the addition of methanol. ZNC-1000 presented no obvious variation after the addition of methanol which demonstrates the excellent toleration for methanol, thus indicates a choice as the electrocatalyst for fuel cells.

## Conclusion

Well designed nanosized ZIF-8 MOF precursor with uniform crystal size of about 78.6 nm has been successfully obtained via combining ultrasonic dispersion treatment with room temperature solution reaction, which is the key to prepare the hierarchical nanoporous N-doped carbon ZNC-1000 ORR electrocatalyst through high temperature pyrolysis. The ZNC-1000 with ~5 at.% N in the framework possesses large surface areas leading to sufficient exposed electrochemical active sites as well as moderate graphitization degree and suitable nanosized hierarchical porosity distributions resulting in the sufficient interaction between O_2_ and the electrocatalyst surface and benefitting the transports of electrons and the electrolyte ions for ORR. The ORR electrocatalytic property of ZNC-1000 is obviously superior to that of the state of art commerical Pt/C in term of onset/half-wave potentials and limiting current density simultaneously. Besides, the stability of ZNC-1000 is much better than that of Pt/C. Thus, ZNC-1000 becomes one of the best ORR electrocatalysts among the MOF-derived non-precious metal nanocatalysts^24^. The refined preparing technique and non-precious metal ORR electrocatalyst are important to the development of fuel cells, metal oxygen batteries and so on.

## Methods

### Materials preparation

#### Preparation of ZIF-8 nanoparticles precursor

ZIF-8 nanoparticles precursor was synthesized via combining ultrasonic dispersion treatment with room temperature solution reaction. Typically, 48 mmol (3.94 g) of 2-methylimidazole was first dissolved in 40 mL of ethanol. Then, a mixture of 12 mmol (3.57 g) of zinc nitrate hexahydrate (Zn(NO_3_)_2_·6H_2_O) and 120 mL of ethanol was added dropwisely into the above solution with stirring, in which the molar ratio of Zn/MeIm/EtOH was 1/4/233. After that, the mixture was treated under ultrasonic for 1 h and further stirred vigorously for 12 h at room temperature. At last, the ZIF-8 nanoparticles were obtained by centrifugation and washed with ethanol for several times.

#### Preparation of the ZNC-x electrocatalyst

The resultant ZIF-8 nanoparticle precursor was heated at 200 °C for 1 h and then increase to the given temperature for 3 h and cooled down to room temperature naturally. The heating process was under flowing N_2_ with the heating rate of 3 °C min^−1^. The obtained nanoporous materials were named as ZNC-x, *i.e*., ZIF-8 derived nitrogen-doped nanoporous carbon with the pyrolysis temperature of x (x = 900, 1000 and 1100) for 3 h. The ZNC-1000 sample presented the best ORR electrocatalytic properties as discussed later. Accordingly, the ZNC-1000–1, 7 and 10 samples were also obtained at 1000 °C with the pyrolysis time of 1, 7 and 10 h, respectively, for comparation. All samples were used for the electrochemical measurement directly without any further wash with acid.

### Materials characterization

SEM measurements were carried out on the HITACHI S-4800F with the operating voltage of 5 kV. (HR)TEM measurements were proceeded on the JEOL JEM-2100F. XRD patterns were obtained from the Panalytical X’Pert Pro X-ray Powder Diffractometer with Cu-Kα radiation. Raman spectroscopy analyses were executed from the LabRAM of Horiba Jobin Yvon. N_2_ adsorption-desorption isotherms were determined from the Micromeritics ASAP 2020 Plus. The specific surface areas were obtained based on the BET method and the pore sizes were calculated based on the DFT method. And XPS analyses were conducted on the ESCALAB 250Xi of Thermofisher.

### Electrochemical measurements

All electrochemical measurements were performed in a three-electrode system on an electrochemical workstation (Pine Instrumentation, Wavedriver 20). The catalysts dispersed on the glassy carbon (GC) rotating disk electrode and rotating ring and disk electrodes (RDE and RRDE, Pine Instrumentation) were performed as the work electrodes with the geometric areas of 0.196 and 0.247 cm^2^, respectively. Ag/AgCl (saturated KCl) and Pt wire were used as the reference and counter electrodes, respectively. To prepare the work electrode, 5.0 mg of the catalyst was suspended in 1.0 mL of the mixture of deionized water, ethanol and 5% of Nafion aqueous solution (v/v/v = 6/3/1) by sonication for 1 h. Then the suspension was dropped by pipette onto the GC electrode uniformly which had been polished with Al_2_O_3_ powder and dried at room temperature. The loaded electrocatalyst mass was 0.32 mg cm^−2^ for all electrodes.

CV curves were conducted at the scan rate of 10 mV s^−1^ from −1.0 to 0 V in N_2_ and O_2_-saturated 0.1 M KOH, respectively. LSV measurements at the scan rate of 10 mV s^−1^ were performed on the RDE and RRDE with different rotation rates (400, 800, 1200, 1600 and 2000 rpm) in O_2_-saturated 0.1 M KOH. The disk potential was from −1.0 to 0 V vs Ag/AgCl and the ring potential was set as 0.6 V vs Ag/AgCl. The ADT were measured at the scan rate of 100 mV s^−1^ for 5000 cycles from −0.4 to 0.8 V vs Ag/AgCl in 0.1 M KOH. The CA method was used to perform the stability at −0.5 V vs Ag/AgCl in O_2_-saturated 0.1 M KOH with the rotating rate of 1600 rpm.

For the RRDE measurement, the four-electron selectivity of the catalysts was evaluated base on the H_2_O_2_ yield as follows:

1 $${{\rm{H}}}_{2}{{\rm{O}}}_{2} \% =200\ast {{\rm{I}}}_{{\rm{R}}}/{\rm{N}}/({{\rm{I}}}_{{\rm{D}}}{+{\rm{I}}}_{{\rm{R}}}/{\rm{N}})$$

2 $${\rm{n}}=4\ast {{\rm{I}}}_{{\rm{D}}}/({{\rm{I}}}_{{\rm{D}}}+{{\rm{I}}}_{{\rm{R}}}/{\rm{N}})$$

where I_D_ is the disk current, I_R_ is the ring current, N is the current collection efficiency of the Pt ring and n is the number of transferred electrons (N = 0.37).

## Electronic supplementary material

Supplementary Information
