# Supplementary material for: Ultrahigh Oxygen Reduction Reaction Electrocatalytic Activity and Stability over Hierarchical Nanoporous N-doped Carbon
Source: Sci Rep. 2018 Feb 12;8:2863. doi: 10.1038/s41598-018-21213-3 (PMC5809453; doi:10.1038/s41598-018-21213-3)
Supplement: Supplementary file 1 — Supplementary Information [file 41598_2018_21213_MOESM1_ESM.doc]

Supplementary Information

**Ultrahigh Oxygen Reduction Reaction Electrocatalytic Activity and Stability over Hierarchical Nanoporous N-doped Carbon**

Zeyu Li^1,2^, Qiuming Gao^1*^, Weiwei Qian^1^, Weiqian Tian^1^, Hang Zhang^1^, Qiang Zhang^1^ & Zhengping Liu^2*^

^1^Key Laboratory of Bio-inspired Smart Interfacial Science and Technology of Ministry of Education, Beijing Key Laboratory of Bio-inspired Energy Materials and Devices, School of Chemistry, Beihang University, Beijing 100191, P. R. China.

^2^Institute of Polymer Chemistry and Physics of College of Chemistry, BNU Lab of Environmentally Friendly and Functional Polymer Materials, Beijing Normal University, Beijing 100875, P. R. China.

^*^Corresponding Authors: [qmgao@buaa.edu.cn](mailto:qmgao@buaa.edu.cn) (QMG) and [lzp@bnu.edu.cn](mailto:lzp@bnu.edu.cn) (ZPL).


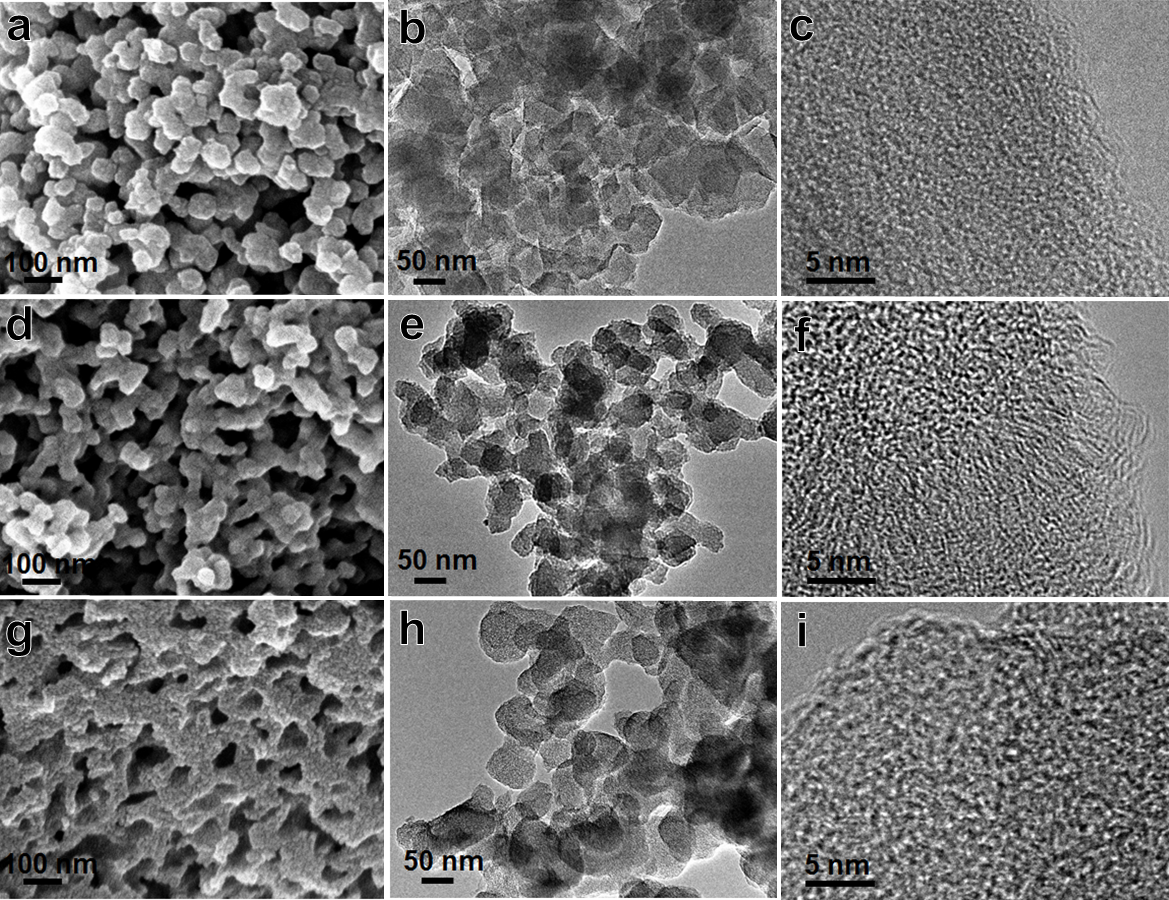


**Figure S1.** SEM, TEM and HRTEM images of ZNC-900 (a-c), ZNC-1000 (d-f) and ZNC-1100 (g-i).

**Figure S2**. XPS survey scan spectra of ZNC-900, ZNC-1000 and ZNC-1100.

**Figure S3**. The CV curves of the ZNCs and the commercial Pt/C in N_2_ and O_2_-saturated 0.1 M KOH, respectively.

**b**

**a**

**c**

**d**

**Figure S4.** The rotation rate-dependent ORR polarization curves for the commercial Pt/C (a) as well as the ZNC-900 (b), ZNC-1000 (c) and ZNC-1100 (d) samples, respectively.

**a**

**b**

**c**

**Figure S5.** The ORR polarization curves for the commercial Pt/C (a) and ZNC-1000 (b) measured at different temperatures with the rotating rate of 1600 rpm in O_2_ saturated 0.1 M KOH. And (c) the Arrhenius plots for the ORR at an overpotential of 0.3 V with the rotating rate of 1600 rpm.

**Table S1.** The content (at.%) of elements derived from EDX.

| Element | ZNC-900 | ZNC-1000 | ZNC-1100 |
| --- | --- | --- | --- |
| C | 80.16 | 91.69 | 94.26 |
| N | 13.38 | 4.65 | 1.83 |
| O | 3.40 | 3.03 | 3.71 |
| Zn | 3.07 | 0.63 | 0.19 |

**Table S2.** The content (at.%) of elements and the bond type (%) related to N derived from the XPS spectra in Fig. S2 and Fig. 2c and d.

| Sample | C | N | O | Zn | Pyridinic N | Pyrrolic N | Graphitic N | Oxidized N | Pyridinic N + Graphitic N |
| --- | --- | --- | --- | --- | --- | --- | --- | --- | --- |
| ZNC-900 | 81.71 | 10.15 | 5.80 | 2.34 | 37.5 | 13.7 | 13.7 | 6.1 | 51.2 |
| ZNC-1000 | 89.11 | 4.72 | 5.73 | 0.44 | 32.1 | 22.2 | 22.6 | 14.9 | 54.7 |
| ZNC-1100 | 94.2 | 2.44 | 3.13 | 0.23 | 19.8 | 18.8 | 25.0 | 28.6 | 34.8 |

**Table S3.** The pore characteristics of the ZNC-900, ZNC-1000 and ZNC-1100 samples.

| Sample | BET surface area  (m^2^ g^-1^) | Micropore surface area^a^  (m^2^ g^-1^) | Mesopore surface area^a^  (m^2^ g^-1^) | Macropore surface area^a^  (m^2^ g^-1^) | Pore volume^a^  (cm^3^ g^-1^) | Micropore volume^a^  (cm^3^ g^-1^) | Mesopore volume^a^  (cm^3^ g^-1^) | Macropore volume^a^  (cm^3^ g^-1^) |
| --- | --- | --- | --- | --- | --- | --- | --- | --- |
| ZNC-900 | 1114 | 1030 | 11 | 17 | 1.23 | 0.36 | 0.17 | 0.71 |
| ZNC-1000 | 1205 | 1035 | 18 | 12 | 1.05 | 0.39 | 0.13 | 0.53 |
| ZNC-1100 | 1076 | 1006 | 19 | 12 | 1.07 | 0.36 | 0.26 | 0.44 |

^a^ Determined by the DFT method.
